# Quercetin Protects Goat Sperm Motility by Inhibiting Neutrophil Extracellular Traps and Maintaining Plasma Membrane and Acrosome Integrity

**DOI:** 10.3390/vetsci11110553

**Published:** 2024-11-10

**Authors:** Zhengkai Wei, Hongrong Hong, Wei Liu, Kaifeng He, Jiaxuan Wang, Xin Guo, Dezhi Zhang, Qianyong Li, Zhengtao Yang

**Affiliations:** 1 College of Veterinary Medicine, Southwest University, Chongqing 400715, China; 2 School of Animal Science and Technology, Foshan University, Foshan 528225, Chinaliu-wei@webmail.hzau.edu.cn (W.L.)

**Keywords:** neutrophil extracellular traps, quercetin, sperm motility, acrosome, plasma membrane

## Abstract

Enhancing or protecting sperm motility has always been a pivotal approach to improving the ewe pregnancy rate. Sperm motility is highly susceptible to the immune status of the reproductive tract. Neutrophil extracellular traps (NETs) have been demonstrated to capture sperm and impair its motility in human, swine, and goat species. Quercetin is a flavonoid derived from *Cuscuta Chinensis Lam*., which can protect sperm from oxidative damage. In this study, we investigated whether inflammation decreases sperm motility and tried to clarify the potential protective mechanism of quercetin on goat sperm motility. Sperm-triggered NETs were analyzed by immunofluorescence analysis. Sperm acrosome integrity was detected by using giemsa staining. Quercetin exhibited no cytotoxicity towards sperm and PMNs within the concentration range of 20–80 μM. PMNs impaired both the survival rate and rapid linear motility of sperm, while quercetin significantly enhanced these parameters. PMNs captured sperm through NETs composed of DNA, citrullinated histone 3 (citH3), and neutrophil elastase (NE); however, quercetin effectively inhibited the release of sperm-stimulated NETs. The stimulation of PMNs with sperm resulted in a significant increase in levels of ROS and MDA, which decreased by quercetin. Moreover, PMNs caused integrity violation to both the plasma membrane and acrosome in sperm; this effect was significantly alleviated by quercetin. In conclusion, quercetin effectively ameliorated PMN-reduced sperm motility through the inhibition of NETs and oxidative stress, and preserving sperm plasma membrane and acrosome integrity, thereby providing preliminary insights into the underlying mechanisms and theoretical support for the development of potential sperm protectors.

## Simple Summary

This study explores the impact of inflammation on sperm motility of goats, and investigates the potential protective role of quercetin, a natural compound, in preserving sperm functionality. Sperm motility is essential for successful fertilization, yet it is susceptible to impairment by inflammation. Neutrophils, key cellular mediator in the inflammatory responses, which compromise sperm through releasing NETs, phagocytosis and induction of oxidative stress. Quercetin, derived from the plant *Cuscuta Chinensis*, is known for its antioxidant properties and ability to protect sperm from oxidative damage. In this study, it was found that neutrophils, when activated by sperm, significantly decreased sperm motility and integrity. However, quercetin treatment effectively inhibited the formation of NETs and reduced oxidative stress, helping to preserve sperm motility and the integrity of sperm’s plasma membrane and acrosome. The results showed that quercetin had no harmful effects on sperm or neutrophils and improved sperm survival and motility. These findings suggest that quercetin could be developed as a potential sperm protector, offering a new approach to improving fertility rates in livestock by mitigating the detrimental effects of inflammation and oxidative stress on sperm quality.

## 1. Introduction

In mammals, successful fertilization requires traversing the female reproductive tract (FRT), which functions as a protective mucous barrier. Approximately 4 billion bull sperm undergo fertilization during natural mating and more than 80% of these sperm are eliminated due to vaginal secretions. Artificial insemination (AI) is widely used in animal husbandry due to the strict storage conditions for frozen semen and its contribution to scientific and technological advancements. However, the freezing process directly damages sperm structure, leading to decreased viability and a low fertilization success rate. The suboptimal quality of thawed ram semen and its limited ability to traverse the tortuous cervix [1,2] lead to reduced pregnancy rates when frozen semen is deposited near the external orifice of the cervix [3]. Sperm are allogeneic to females and may encounter the female immune system’s defense against infectious organisms [4]. During natural conception or AI, pathogenic bacteria can enter the female reproductive tract alongside sperm, eliciting an immune response and recruiting a substantial number of PMNs to the reproductive tract mucosa. These PMNs possess the capability to capture, degrade, and eliminate pathogenic bacteria through NETs. However, this process can also exert detrimental effects on sperm. Therefore, enhancing sperm quality will be a brilliant solution [5,6].

Various factors, such as uterine wall grooves and adverse physical environments, exert an influence on the motility and function of goat sperm. Sperm damage is a result of environmental fluctuations, osmotic and oxidative changes, catabolic alterations, the interplay between uterine muscle contractions and mucus secretion, and neutrophil phagocytosis [7,8]. The vagina possesses a robust antibacterial defense mechanism against external factors, encompassing an immune response that may potentially impair sperm viability and combat infectious agents [4]. Polymorphonuclear neutrophils (PMNs) are essential components of the innate immune system and inflammation, constituting the first line of defense against infectious pathogens. PMNs directly attack foreign objects through phagocytosis, secretion of reactive oxygen species (ROS), and release of neutrophil extracellular traps (NETs). NETs primarily consist of DNA, citrulline histone 3 (CitH3), myeloperoxidase (MPO), and neutrophil elastase (NE) [9,10,11]. ROS are essential byproducts of aerobic metabolism and play crucial roles in various physiological processes, including embryonic development, gamete maturation, and fertilization [12,13]; however, they can also have detrimental effects on boar [14]. In cattle [15], pigs [16], and horses [17], compelling evidence suggests that PMNs engulf sperm and infiltrate the uterine cavity shortly after fertilization. The release of sperm in the female reproductive tract is believed to primarily elicit the PMNs responses [10,15,16,18]. In vitro and in vivo studies have demonstrated that PMNs selectively phagocytose viable sperm cells [18]. Therefore, the identification of sperm-protective agents that mitigate neutrophil-induced damage and enhance sperm motility represents a pivotal focus in the development of semen-preserving diluents.

**Figure 1:**
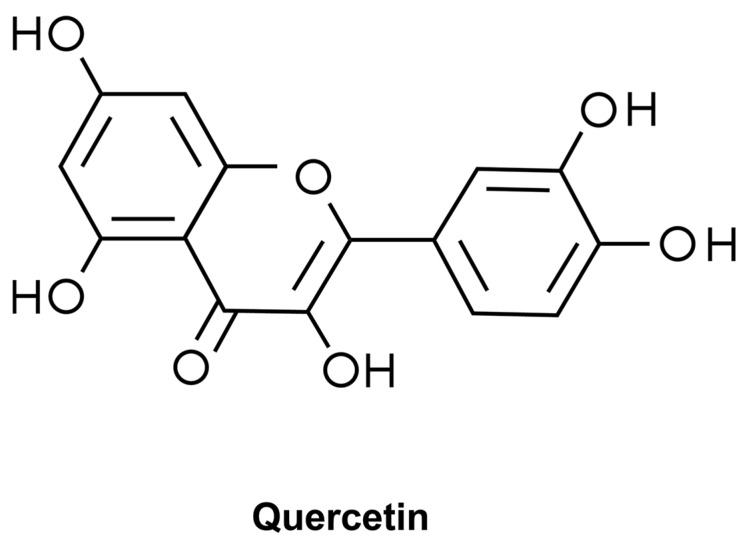
Chemical structure of quercetin.

*Cuscuta Chinensis Lam.* is employed as a botanical agent in the clinical management of male infertility. The investigated plant contains a diverse range of bioactive compounds, including flavonoids, steroids, volatile oils, lignans, polysaccharides, alkaloids, and other constituents as documented in previous scientific investigations [19,20,21]. Quercetin, a flavonoid compound as depicted in Figure 1, is a constituent of *Cuscuta chinensis Lam.* and is also found in a variety of fruits, vegetables, and leaves. This compound is frequently used to address male infertility, given its efficacy in alleviating structural and functional impairments caused by oxidants in sperm [22]. Its remarkable antioxidant properties have attracted significant attention in cancer research [23] and gliotoxin poisoning [24]. Quercetin has been demonstrated to alleviate oxidative stress by scavenging free radicals, thereby protecting sperm from ROS-induced damage [25]. Studies have demonstrated that quercetin supplementation can enhance sperm motility and viability or reduce lipid peroxidation in various species, including humans [26], rabbit [27], and buffalo [28]. Additionally, quercetin may also prevent the release of extracellular enzymes that contribute to sperm cell damage during processes like cryopreservation [27,28]. This antioxidant’s protective role makes it a promising agent in improving male fertility and preserving sperm quality under stress conditions [29]. Sperm is particularly susceptible to oxidative stress due to its limited antioxidant enzyme activity. A study conducted on rats has demonstrated that quercetin can restore testicular function and revitalize sperm production through its antioxidant properties [30]. However, it remains unclear whether quercetin has similar effects on goat sperm. Despite their potential economic benefits, low conception rates remain a challenge in goat breeding. Therefore, we selected goat sperm as our research subject to investigate the protective effects and mechanism of quercetin on sperm motility.

## 2. Materials and Methods

### 2.1. Materials and Reagents

Quercetin, the Goat Peripheral Blood Neutrophil Isolation Kit^®^, and glutamine-free RPMI Medium 1640 containing glucose were purchased from Beijing Solarbio (Beijing Solarbio Science & Technology Co., Ltd., Beijing, China). Deoxyribonuclease I (DNase I), 2,7-dichlorofluorescein diacetate (DCFH-DA), and Zymosan (58856-93-2) were purchased from Sigma-Aldrich (St. Louis, MO, USA). The MDA Assay Kit, Glutathione Peroxidase (GSH-PX) Assay Kit, CCK-8 Assay kit, and CAT Assay Kit were purchased from Nanjing Jiancheng Institute of Biological Engineering (Nanjing, China).

### 2.2. Isolation of Goat PMNs

Adult female black goats were provided with a ration for the purpose of maintenance. Blood was collected from the jugular vein and diluted 6-fold with glutamine-free RPMI Medium 1640 containing glucose. Following the protocol described in the manuscript of the Goat Peripheral Blood Neutrophil Isolation Kit^®^ (Solarbio, Beijing, China), we isolated polymorphonuclear cells (PMNs) from goat blood. Experiments on animals were approved by the Animal Ethics Committee of Foshan University, China (FOSU2022030).

### 2.3. Thawing Frozen Goat Sperm

Boer goat sperm (Hualin Animal Husbandry, Xinjiang, China) were obtained from the liquid nitrogen, quickly rewarmed at 37 °C for 2–5 min, and equilibrated at room temperature for 10 min. The semen exhibited normal odor and color, with a viability of approximately 80% at room temperature. Subsequently, sperm was centrifugated at 650× *g* for 20 min and resuspended in glutamine-free RPMI Medium 1640 containing glucose.

### 2.4. Cell Counting Kit-8 Assay

PMNs (2 × 10^5^ cells/well) were seeded onto 96-well culture plates and incubated for 30 min, followed by pretreatment with quercetin (20, 40, 80 μM) for 30 min. Subsequently, the cells were co-incubated with goat sperm (1:3) and quercetin (20, 40, 80 μM) for a duration of 90 min. CCK-8 reagent was then added and incubated for a period of 2 h. The absorbance was measured at a wavelength of 450 nm.

### 2.5. Goat Sperm Viability Measurement

PMNs (10^6^ cells/well) were added to 24-well culture plates and incubated for 30 min, followed by pretreatment with quercetin (20, 40, 80 μM) for an additional 30 min. Subsequently, the cells were co-incubated with goat sperm (1: 3) and quercetin (20, 40, 80 μM) for varying durations of 1 h, 1.5 h, 3 h, and 6 h to assess sperm viability. Sperm viability (sperm survival rate) = the number of linear sperm (survival sperm) / total sperm movements counted ×100%. For analysis purposes, 10 μL of aspirated samples was placed on slides and covered with coverslips prior to microscopic observation at a magnification (200×) in five different fields of view, each containing a minimum count of 100 sperm.

### 2.6. Quantification of NETs

PMNs were co-incubated with goat sperm (1:3) and quercetin (20, 40, 80 μM), and sperm-induced NETs were quantified by using a Pico Green^®^ (Invitrogen, Carlsbad, CA, USA) and Infiniti M200^®^ (TECAN, Hombrechtikon, Switzerland) fluorescence microplate reader at an excitation wavelength of 485 nm and emission wavelength of 535 nm.

### 2.7. Immunofluorescence Analysis

PMNs (10^6^ cells/well) were cultured on slides and stimulated with goat sperm (1:1), quercetin, and Zymosan (1 mg/mL) for 90 min. Subsequently, the cells were fixed with 4% paraformaldehyde for 20 min. Following fixation, the slides were washed with sterile phosphate-buffered solution (PBS), permeabilized with 1% Triton X-100 for 15 min, and rinsed three times with PBS. To block nonspecific binding sites, a solution of 3% goat serum was applied to seal the cells for 3 h. The sample was then incubated overnight at 4 °C with anti-elastase antibody (diluted at 1:300; ab68672, Abcam, Cambridge, UK) and anti-citH3 antibody (diluted at 1:300; ab5103, Abcam, Cambridge, UK). Finally, the cells were incubated with a secondary antibody (diluted at 1:100; SA00003, Proteintech, Chicago, USA) for 2 h and stained with Sytox Orange (5 μM) for a duration of 15 min. Fluorescence microscopy was employed to capture images.

### 2.8. ROS Production Assay

ROS levels was quantified using DCFH-DA (Sigma-Aldrich, St. Louis, MO, USA). After incubation of PMNs, sperm, and quercetin as previously described, DCFH-DA was added and incubated for 30 min. Fluorescence intensity was obtained using an Infiniti M200^®^ microplate reader (TECAN, Hombrechtikon, Switzerland).

### 2.9. Analysis of Catalase (CAT), Glutathione Peroxidase (GSH-PX), and MDA

PMNs (2 × 10^5^ cells/well) were seeded into 96-well culture plates and incubated for 30 min, followed by pretreatment with quercetin (20, 40, 80 μM) for an additional 30 min. Subsequently, the cells were stimulated with goat sperm (1:3) for a duration of 90 min. Afterward, RIPA Lysis Buffer (200 μL) was added to the plates and allowed to lyse for a period of 10 min. The cell lysis solution was centrifuged at a speed of 12,000 rpm/min for 10 min at a temperature of 4 °C. CAT catalyzes the breakdown of hydrogen peroxide (H₂O₂), with the reaction being rapidly halted by the addition of ammonium molybdate. The residual H₂O₂ reacts with ammonium molybdate to form a yellowish complex. Glutathione peroxidase (GSH-PX) facilitates the reaction of H₂O₂ with reduced glutathione (GSH), producing water (H₂O) and oxidized glutathione (GSSG). Malondialdehyde (MDA), a product of lipid peroxidation, reacts with thiobarbituric acid (TBA) to form a red complex, which exhibits a maximum absorbance at 532 nm. According to the manufacturer’s instructions, CAT and GSH-PX activity was measured using a CAT and GSH-PX Assay Kit at 405 nm, while MDA levels were measured at 532 nm.

### 2.10. Goat Sperm Acrosome Integrity Test

The acrosome integrity rate was assessed using Giemsa staining. PMNs (10^6^ cells/well) were seeded onto 24-well culture plates for a duration of 30 min and pre-treated with quercetin (20, 40, 80 μM) for an additional 30 min. Subsequently, the cells were co-incubated with goat sperm (1:3) and quercetin (20, 40, 80 μM). After incubation for 1 h, a volume of 10 μL from each sample was dropped onto a slide and fixed with a solution containing 4% paraformaldehyde for a period of 30 min. The slides were then washed with PBS and air-dried before being stained with Giemsa staining solution for 2 h. Following another round of PBS washing and natural drying, the images were observed under a microscope. Sperm acrosome integrity rate = several sperm with entire acrosome / total number of sperm ×100%.

### 2.11. Determination of Goat Sperm Plasma Membrane Integrity

The tail bending rate of sperm is a measure of the integrity of the sperm plasma membrane. Hypotonic swelling causes the tails of sperm to bend. The tail turning rate of sperm = the number of sperm with bent tails/total sperm counted ×100%. PMNs (10^6^ cells/well) were incubated in 96-well culture plates for 30 min and pretreated with quercetin (20, 40, 80 μM) for an additional 30 min. Subsequently, these cells were co-incubated with goat sperm (1:3) and quercetin (20, 40, 80 μM) for 1 h. A hypotonic solution was prepared in a centrifuge tube at a temperature of 37 °C for 5 min; then, samples (100 μL) were added to this solution and maintained at a temperature of 37 °C for 30 min. Finally, ten microliters (10 μL) from each sample were aspirated onto a slide and covered with a coverslip before microscopic observation in five different fields.

### 2.12. Statistical Analysis

The statistical analysis was performed using GraphPad Prism 7 (San Diego, CA, USA). A one-way analysis of variance (ANOVA) and a two-way analysis of variance were conducted to compare the groups, followed by Tukey’s post hoc test. The values are expressed as means ± SEM. Statistical significance is denoted as “*ns*” for not significant; * *p* < 0.05, ** *p* < 0.01, and *** *p* < 0.001.

## 3. Results

### 3.1. Quercetin Exhibited No Cytotoxic Effects on Goat Sperm and PMN

**Figure 2:**
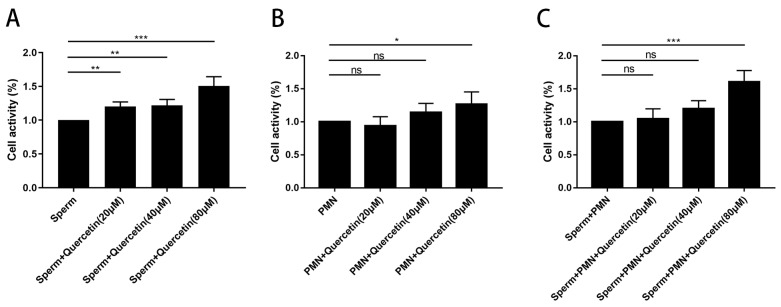
Effects of quercetin on sperm and PMNs activity. (**A**) Effects of quercetin on sperm activity. Quercetin exhibited no cytotoxic effects on sperm. (**B**) Effects of quercetin on PMNs activity. Quercetin did not influence the PMNs activity. (**C**) Effects of quercetin on sperm and PMNs activity. Quercetin showed non-toxicity to both PMNs and sperm. The values are presented as means ± SEM (n = 5, “*ns*” signifies not significant; * *p* < 0.05, ** *p* < 0.01, *** *p* < 0.001).

Co-incubation of quercetin at concentrations of 20, 40, and 80 µM with PMNs and goat sperm indicated that quercetin did not induce cytotoxic effects on either goat sperm or PMNs. Notably, these concentrations of quercetin significantly enhanced cell activity in both goat sperm (Figure 2A) and PMNs (Figure 2B), as well as in the combined group of goat sperm and PMNs (Figure 2C).

### 3.2. Quercetin Enhanced Goat Sperm Survival and Motility

**Figure 3:**
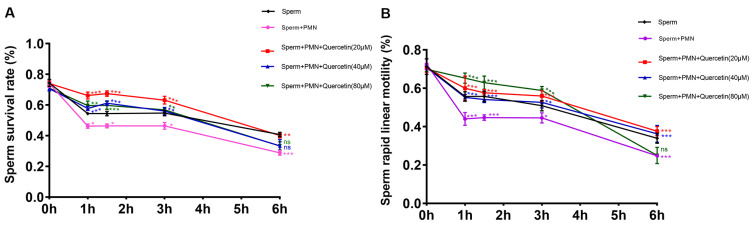
Effects of quercetin on goat sperm survival and rapid linear motility. (**A**) Effects of quercetin on goat sperm survival rate. PMNs significantly reduced the sperm survival rate, but quercetin at concentrations of 20, 40, and 80 μM increased the sperm survival rate decreased by PMNs at 1, 1.5, and 3 h. Notably, only quercetin at 20 μM continued to provide protection at 6 h. (**B**) Effects of quercetin on goat sperm’s rapid linear motility. PMNs significantly reduced sperm’s rapid linear motility. Quercetin at 20 and 40 μM was able to increase the sperm’s rapid linear motility that decreased by PMNs at 1, 1.5, 3, and 6 h. The values are presented as means ± SEM (n = 5, “*ns*” signifies not significant; * *p* < 0.05, ** *p* < 0.01, *** *p* < 0.001).

The rapid linear motility of goat sperm is widely recognized as a crucial indicator of sperm quality. We observed a significant reduction in sperm viability and rapid linear motility following exposure to PMNs, whereas quercetin treatment resulted in a pronounced enhancement in both parameters compared to the control group (Figure 3A,B).

### 3.3. Goat-Sperm-Induced NETs Composed of DNA, citH3, and NE

**Figure 4:**
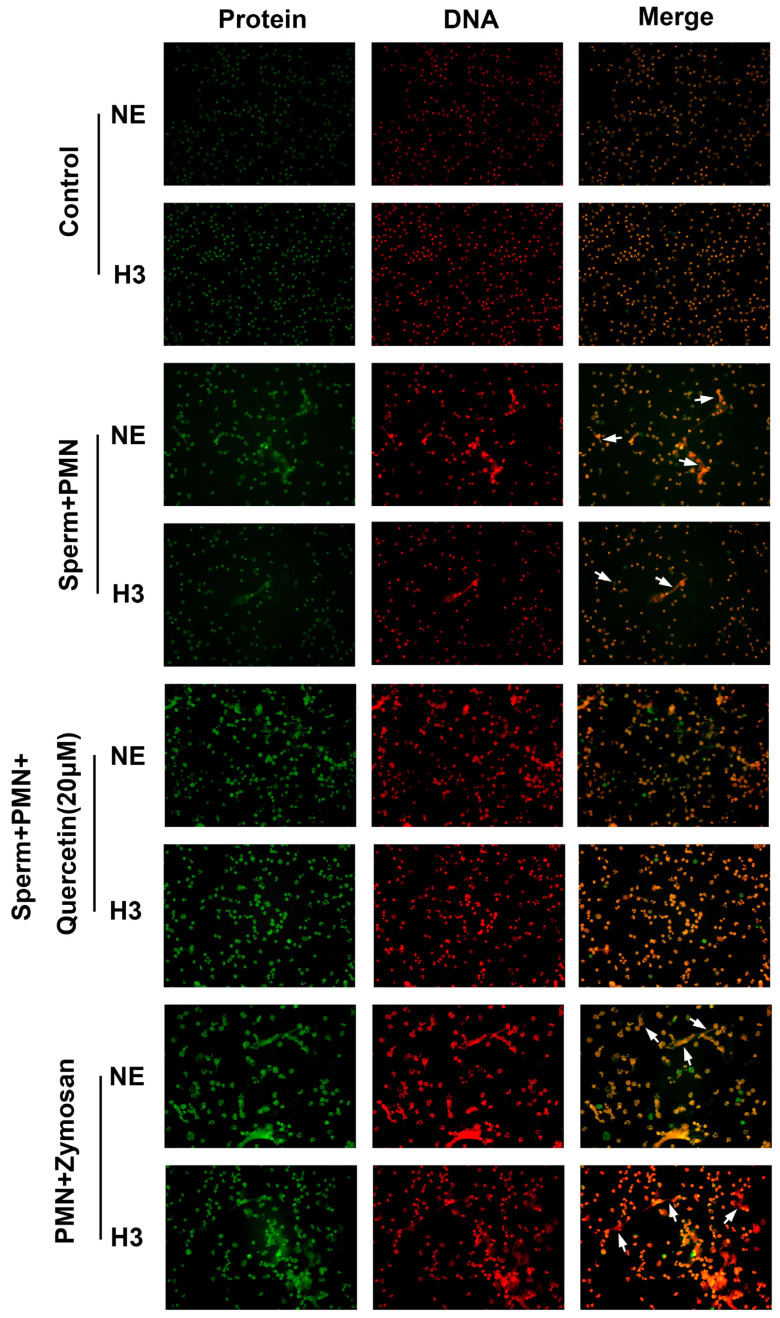
Visualization of quercetin on sperm inducing the formation of NETs. Goat sperm triggered the production of NETs, characterized by DNA scaffolds (Red) decorated with citH3 (Green) and NE (Green), and quercetin-reduced sperm induced the formation of NETs (white arrows).

Goat sperm were found to trigger the release of NETs, characterized by DNA decorated with citH3 and NE, similar to Zymosan and quercetin at a concentration of 20 µM significantly that decreased the formation of NETs (Figure 4).

### 3.4. Quercetin Reduced the NETs Triggered by Goat Sperm

**Figure 5:**
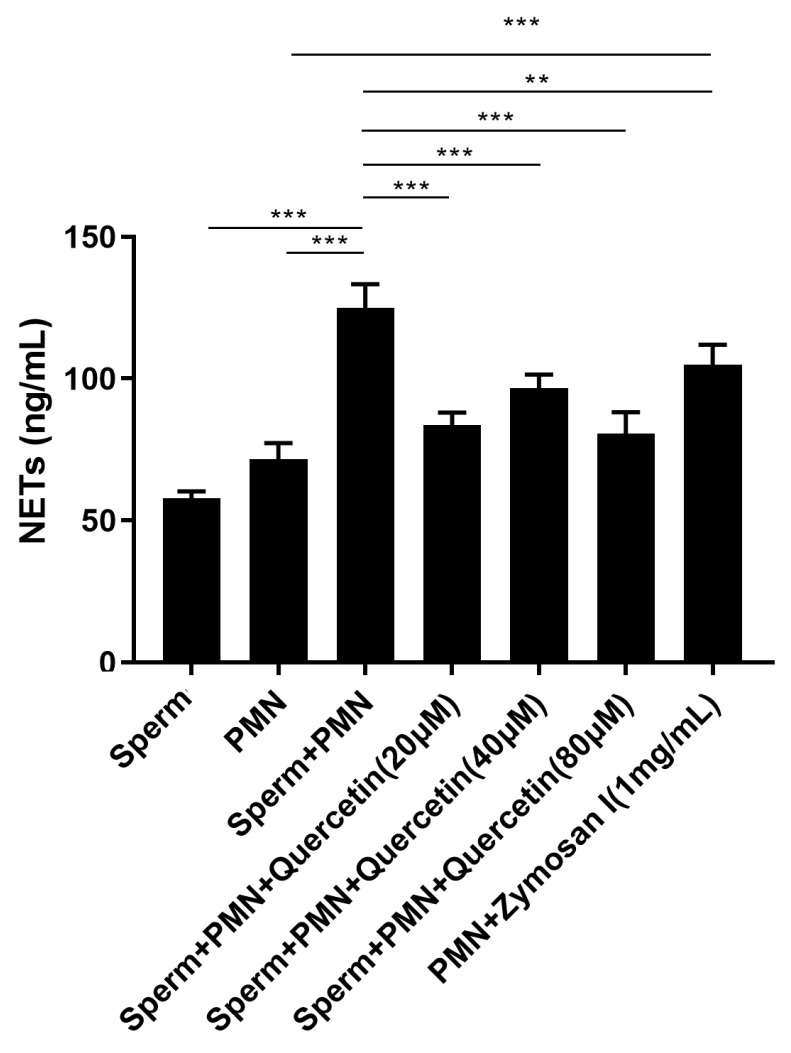
Quantitation of quercetin on sperm-induced NETs. Zymosan (1 mg/mL, Sigma) served as the positive control group. Quercetin effectively reduced NET production stimulated by sperm compared to the sperm and PMNs group, demonstrating that quercetin could decrease sperm-induced NET release. The values are presented as means ± SEM (n = 5; ** *p* < 0.01, *** *p* < 0.001).

Quantitative analysis of sperm-triggered NETs revealed that the levels of NETs were significantly higher in the sperm and PMNs mixture compared to either sperm or PMNs alone, even surpassing the levels induced by Zymosan as a positive control. Importantly, quercetin effectively reduced the production of NETs stimulated by sperm (Figure 5).

### 3.5. Quercetin Effectively Inhibited NETs by Attenuating ROS Production

**Figure 6:**
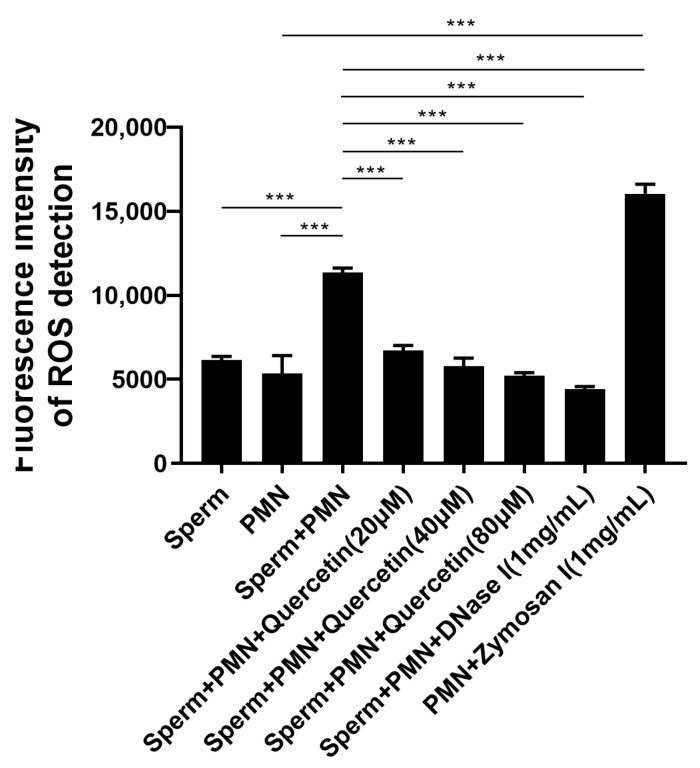
Quercetin effectively inhibited NETs by attenuating ROS production. Zymosan (1 mg/mL, Sigma) served as the positive control group. Sperm or Zymosan significantly increased ROS production compared to PMNs. Treatment with quercetin significantly attenuated ROS generation compared to the sperm–PMNs mixture group. The values are presented as means ± SEM (n = 5; *** *p* < 0.001).

ROS levels in the sperm and PMNs group mixture were significantly increased compared to sperm alone or PMNs alone, but lower than that induced by Zymosan. DNase I, used as a standard degrader of NETs both in vitro and in vivo, significantly decreased sperm-induced ROS levels in PMNs. Treatment with quercetin significantly attenuated ROS generation compared to both the sperm and PMNs mixture and Zymosan (Figure 6).

### 3.6. Quercetin Protected Goat Sperm by Counteracting Oxidative Stress and Inhibiting Lipid Peroxidation

**Figure 7:**
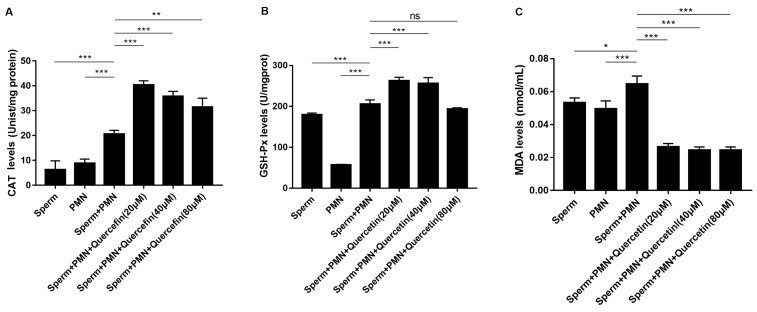
Effects of quercetin on CAT, GSH-Px, and MDA levels. (**A**) CAT levels (n = 3). (**B**) GSH-PX levels (n = 5). (**C**) MDA levels (n = 5). Goat sperm significantly increased CAT, GSH-Px, and MDA levels, while quercetin enhanced CAT and GSH-PX activity and reduced MDA levels. The values are presented as means ± SEM (n = 5, “*ns*” signifies not significant; * *p* < 0.05, ** *p* < 0.01, *** *p* < 0.001).

Oxidative stress is widely recognized to play a pivotal role in sperm damage. We investigated the impact of quercetin on the oxidation state in goat sperm and PMNs. Combined sperm + PMNs resulted in increased CAT levels, indicating enhanced oxidative stress and a compensatory rise in antioxidant defense. Quercetin at concentrations of 20, 40, and 80 µM significantly increased CAT activity compared to the sperm + PMNs group (Figure 7A). Quercetin maintained high GSH-Px levels at 20 and 40 µM, although there was no statistically significant difference at 80 µM (Figure 7B). Furthermore, quercetin at all tested concentrations significantly reduced MDA levels compared with the sperm and PMNs group (Figure 7C).

### 3.7. Quercetin Preserved Goat Sperm by Preserving Its Structural Integrity

**Figure 8:**
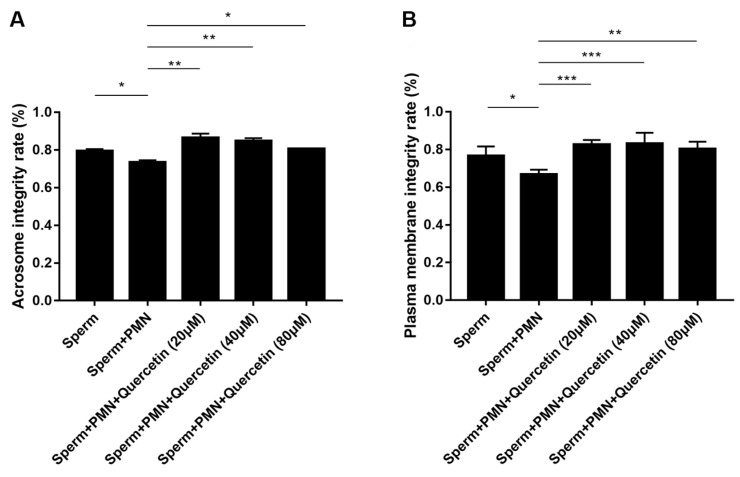
Effects of quercetin on acrosome and plasma membrane integrity. (**A**) Acrosome integrity. (**B**) Plasma membrane integrity. PMNs caused integrity violation to both the plasma membrane and acrosome in sperm; however, this effect was significantly alleviated by quercetin. The values are presented as means ± SEM (n = 5; * *p* < 0.05, ** *p* < 0.01, *** *p* < 0.001).

The structural integrity of goat sperm, reflected by the acrosome and plasma membrane integrity, serves as crucial indicators. Our results demonstrated that PMNs cause integrity violations to both the plasma membrane and acrosome in sperm; however, these effects were significantly alleviated by quercetin at concentrations of 20, 40, and 80 µM (Figure 8).

## 4. Discussion

The diminishing PMN-mediated capture and injury of sperm represents a promising strategy to enhance sperm motility through the female reproductive tract, potentially increasing pregnancy rates. Our study demonstrates that quercetin effectively protects sperm from impairment caused by PMNs, offering preliminary insights into the underlying mechanisms and providing theoretical support for the development of potential sperm protectors.

In this study, quercetin was found to be non-cytotoxic to goat sperm and PMNs, actually enhancing their viability. Oxidative stress is a significant factor in sperm quality decline [31], and quercetin might mitigate this by diminishing free radical production, thus preserving the physiological functions of both sperm and PMNs. Our findings revealed that the presence of PMNs releasing NETs resulted in a decrease in goat sperm viability and motility, aligning with observations in pig and human sperm studies [11,32]. The release of NETs is often coupled with reactive oxygen species (ROS) [33,34], and excessive ROS can significantly damage sperm [35,36], causing membrane peroxidation and DNA damage, ultimately impairing fertility [14]. The release of NETs, serving as an immune defense mechanism, could also have detrimental effects on reproductive cells.

Oxidative stress is a critical factor in the process of sperm damage induced by PMNs. Antioxidant enzymes, such as CAT and GSH-Px, are essential in maintaining cellular redox balance. Conversely, MDA, a product of lipid peroxidation, serves as a reliable biomarker for oxidative stress. It has been linked to harmful biological and biochemical changes in sperm during in vitro preservation [14]. Elevated levels of MDA within cells can initiate biomolecule aggregation, resulting in significant cytotoxic effects. In our study, PMNs stimulation significantly increased MDA levels, indicating that sperm were under considerable oxidative stress due to PMNs activity. Quercetin mitigates this by upregulating GSH-Px and CAT activities and reducing MDA levels, thus alleviating oxidative damage caused by PMNs on goat sperm. The antioxidant effects of quercetin likely stem from its ability to scavenge free radicals, decrease lipid peroxidation, and preserve cellular membrane integrity and other biomolecules, thereby protecting sperm function.

Acrosome and plasma membrane integrity is crucial for assessing sperm quality. Our findings showed that PMNs compromise the integrity of these structures, but quercetin significantly improved acrosome integrity and stabilized the sperm membrane. This suggests that quercetin protects sperm structural integrity through its dual role in antioxidation and inhibiting NET release, thereby enhancing overall sperm quality. This provides strong support for quercetin as a potential sperm quality protector. Quercetin’s ability to preserve sperm membrane and acrosome integrity is critical in preventing the detrimental effects of NETs, such as membrane damage or entrapment in NETs. This highlights quercetin’s protective role in maintaining sperm structure under the oxidative stress associated with NET formation, enhancing reproductive potential.

## 5. Conclusions

The current study underscores the potential of quercetin to effectively protect goat sperm from PMN-mediated damage by degrading NETs, thereby mitigating oxidative injury and preserving structural integrity. This article preliminarily confirms the underlying mechanisms of quercetin in protecting goat sperm from PMN-induced damage, providing robust theoretical support for the potential application of quercetin as a promising sperm protector.

## Data Availability

Data is unavailable due to a pending patent application.
